# Pharmacological mechanisms and clinical evidence of vasopressin, steroids, and epinephrine triple therapy in in-hospital cardiac arrest

**DOI:** 10.3389/fcvm.2026.1912914

**Published:** 2026-08-03

**Authors:** Chong Ma, Tianlong Wang, Yan Zhou, Nan Wang, Chao Lan

**Affiliations:** 1 The First Affiliated Hospital of Zhengzhou University, Zhengzhou, Henan, China; 2 The Fifth Affiliated Hospital of Zhengzhou University, Zhengzhou, Henan, China

**Keywords:** glucocorticoids, in-hospital cardiac arrest, post-cardiac arrest syndrome, return of spontaneous circulation, vasopressin-steroids-epinephrine

## Abstract

In-hospital cardiac arrest carries a hospital discharge survival rate of 22.6% in high-income settings, a figure unchanged over the past decade. Epinephrine reliably promotes return of spontaneous circulation (ROSC) but does not consistently improve neurologically intact survival. This limitation motivated the development of the vasopressin, steroids, and epinephrine (VSE) combination. This review examines the pharmacological rationale underpinning VSE and evaluates the clinical evidence across randomised trials, individual participant data analyses, and network meta-analyses. Vasopressin contributes V1a receptor-mediated vasoconstriction independent of adrenergic receptor status. Intra-arrest methylprednisolone enhances vascular reactivity and attenuates post-ROSC vasopressor requirements. Post-resuscitation hydrocortisone targets critical illness-related corticosteroid insufficiency, constituting a two-phase steroid strategy that defines the full Mentzelopoulos protocol. Pooled analysis of three randomised trials enrolling 869 patients assigns moderate certainty to the ROSC benefit and low certainty to survival and neurological outcome endpoints. A network meta-analysis of 36 trials enrolling 21,768 patients ranked VSE above all comparators for haemodynamic outcomes while leaving neurological endpoints unresolved. Individual patient data reanalysis identifies an independent protective effect of the corticosteroid component against post-resuscitation septic shock. The dissociation between ROSC and neurological recovery reflects insufficient statistical power for hard endpoints, protocol differences between trials, compartmentalised neuroinflammation only partially accessible to circulating glucocorticoids, and epinephrine-mediated cerebral microcirculatory impairment. The 2025 American Heart Association, European Resuscitation Council, and International Liaison Committee on Resuscitation guidelines do not recommend routine VSE during in-hospital cardiac arrest. Adequately powered trial evidence on patient-centred outcomes remains pending. Patient phenotype stratification and factorial trial design represent the most promising directions for future investigation.

## 1 Introduction

In-hospital cardiac arrest (IHCA) remains a major contributor to hospital mortality across acute care settings. Survival to discharge stands at 22.6% in high-income healthcare systems, a figure unchanged over the past decade (1). Return of spontaneous circulation (ROSC) is achieved in roughly two-thirds of resuscitation attempts, yet fewer than one-quarter of those patients ultimately survive to discharge (2). Survival gains observed through 2,010 have since plateaued, and incremental refinements to current protocols appear unlikely to alter this trajectory (3).

Epinephrine has occupied a central position in advanced cardiac life support (ACLS) for decades, primarily through its capacity to augment coronary perfusion pressure and promote ROSC. The PARAMEDIC-2 trial in 8,014 patients with out-of-hospital cardiac arrest confirmed that epinephrine improves both ROSC and 30-day survival compared with placebo (4). However, survivors in the epinephrine group had substantially higher rates of severe neurological impairment at discharge (5). Restoring cardiac output does not resolve the downstream organ injury that determines functional recovery.

The biological basis of this limitation lies in post-cardiac arrest syndrome (PCAS). This multisystem response to global ischaemia-reperfusion encompasses brain injury, myocardial dysfunction, circulatory failure, and pronounced inflammatory activation (6). Immune dysregulation after ROSC further amplifies tissue damage and predisposes survivors to secondary infection (7). Epinephrine engages the acute circulatory failure that precipitates arrest but does not address the downstream mechanisms governing post-resuscitation organ recovery.

The combination of vasopressin, steroids, and epinephrine (VSE) was developed to address these limitations. The regimen administers intravenous vasopressin and methylprednisolone during cardiopulmonary resuscitation (CPR), followed by stress-dose hydrocortisone after ROSC. Two Greek tertiary care trials enrolling 100 and 268 patients reported significant improvements in ROSC and in neurologically favourable survival (8, 9). A subsequent multicentre Danish trial in 501 patients replicated the ROSC benefit but found no improvement in 30-day survival or neurological outcome (10). This null pattern persisted at one-year follow-up (11).

This divergence between consistent ROSC gains and unstable patient-centred endpoints defines the organising question of this review. We examine the pharmacological rationale underpinning VSE, evaluate the clinical evidence across randomised trials, individual participant data analyses, and network meta-analyses, and address why the divergence persists. Safety considerations and directions for future trial design follow from these findings.

## 2 Pharmacological basis of the VSE combination

### 2.1 Limitations of epinephrine monotherapy during prolonged resuscitation

**Figure 1:**
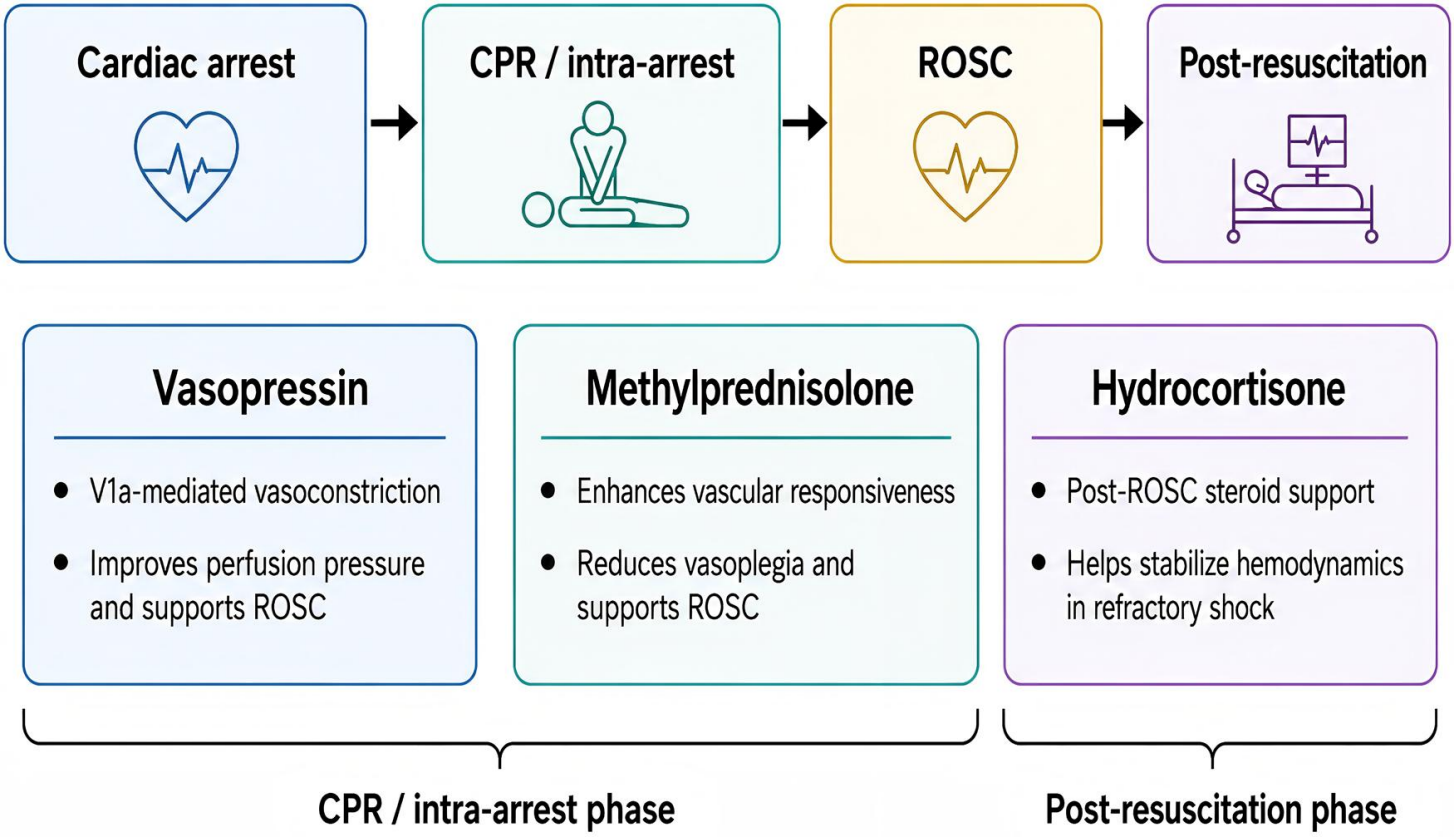
Temporal framework of VSE therapy during iN,hospital cardiac arrest. Vasopressin and methylprednisolone are administered during CPR, followed by post-ROSC hydrocortisone. Vasopressin provides adrenergic-independent vasoconstriction, methylprednisolone enhances vascular responsiveness during CPR, and hydrocortisone supports post-resuscitation haemodynamic stabilisation.

**Figure 2:**
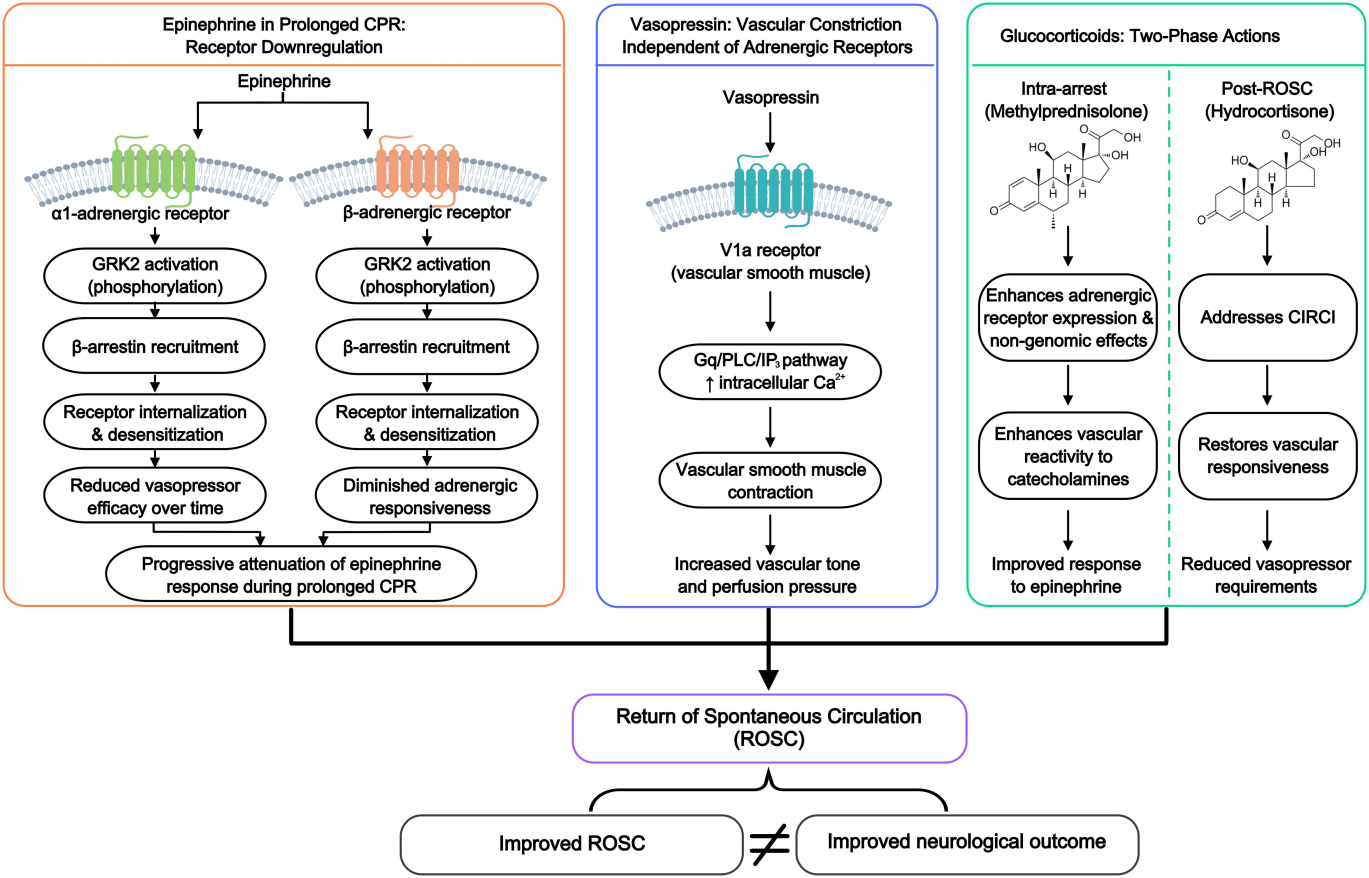
Pharmacological mechanisms underlying the VSE combination during cardiac arrest. Prolonged resuscitation downregulates *α*1- and *β*-adrenergic receptors, which limits the response to epinephrine. Vasopressin restores vascular tone through V1a receptors independently of adrenergic status. Glucocorticoids act across two phases, enhancing adrenergic responsiveness during CPR and addressing CIRCI after ROSC. These mechanisms converge on ROSC, which does not translate uniformly into favourable neurological outcome.

The pharmacological rationale for VSE begins with a physiological problem that epinephrine alone cannot resolve. The temporal and pharmacological architecture of the regimen is summarised in Figure 1. During sustained CPR, repeated administration of high-dose catecholamines progressively desensitises adrenergic receptors. The response to each successive dose is thereby reduced (12). At *α*1-adrenergic receptors on vascular smooth muscle this attenuates vasopressor efficacy over time. The same process affects the β1/β2-adrenergic receptors, particularly the β2 subtype. These receptors mediate several actions that distinguish epinephrine from pure *α*-agonist vasopressors. They support chronotropic and inotropic function in the ischaemic myocardium and facilitate the sarcolemmal sodium-potassium pump that assists restoration of a perfusing rhythm (13). The underlying molecular cascade is shared across both receptor families. It comprises G protein-coupled receptor kinase 2 phosphorylation, *β*-arrestin recruitment, and receptor internalisation, each of which reduces functional surface receptor availability (14) (Figure 2). This mechanistic description derives largely from adrenergic biology in critical illness models rather than from CPR-specific data. Nonetheless, the attenuation is understood as a biological consequence of prolonged adrenergic exposure rather than a dosing artefact.

Critically, this receptor downregulation is not contingent on a septic aetiology. In cardiac arrest it is driven by the intrinsic pathophysiology of the event. The principal drivers are global ischaemia-reperfusion, profound tissue acidosis, and the combined burden of the endogenous catecholamine surge and repeated exogenous epinephrine dosing. This process therefore arises even in IHCA unrelated to sepsis. A mechanistically analogous downregulation is documented in septic shock, where sustained catecholamine surplus limits the haemodynamic response to norepinephrine, and this serves as parallel corroboration (15). These observations establish a biological justification for introducing vasopressor mechanisms that do not depend on adrenergic receptor occupancy. This is the gap the non-epinephrine components of VSE are designed to address. The concurrent *β*-adrenergic desensitisation also provides a rationale for restoring adrenergic receptor responsiveness pharmacologically, a role examined for the corticosteroid component below.

### 2.2 Vasopressin: adrenergic-independent vasoconstriction

Vasopressin addresses this gap through V1a receptor-mediated vasoconstriction. This pathway operates independently of adrenergic receptor status and remains theoretically active in the receptor-attenuated environment of prolonged CPR (16). V1a receptors are broadly expressed across vascular smooth muscle beds, producing generalised vasoconstriction that augments systemic vascular resistance during circulatory collapse. Within the renal circulation, V1a-mediated constriction predominates at the efferent arteriole and helps maintain glomerular filtration pressure under reduced cardiac output (17). V2 receptor activation carries a counterbalancing risk of promoting renal water reabsorption and augmenting capillary permeability (18). Vasopressin's contribution to VSE is therefore most coherent as a time-limited intra-arrest adjunct rather than a sustained post-ROSC infusion, an interpretation reflected in the protocols of both major VSE trials.

### 2.3 Corticosteroids: a two-phase pharmacological strategy

Corticosteroids contribute across two distinct time windows, and each targets a different physiological problem. During CPR, intravenous methylprednisolone is administered with the goal of enhancing vascular reactivity and reducing dependence on exogenous catecholamines. The most clinically grounded evidence for this mechanism comes from the haemodynamic sub-study of the STEROHCA trial. That trial examined prehospital high-dose glucocorticoid administration in patients resuscitated from out-of-hospital cardiac arrest (19). The evidence derives from an out-of-hospital population, and direct extrapolation to IHCA requires caution.

A single prehospital bolus of 250 mg intravenous methylprednisolone produced a significant reduction in cumulated norepinephrine requirement over the 0–48 h post-admission window. The most pronounced separation occurred at 24 h. Mean arterial pressure was consistently higher in the glucocorticoid arm from 6 h onward. Pulmonary artery catheter data from the Rigshospitalet sub-cohort showed no between-group differences in cardiac output, systemic vascular resistance, or mixed venous oxygen saturation. This pattern indicates that the vasopressor-sparing effect was driven by mitigation of vasoplegia rather than by improved myocardial contractility. These analyses were *post-hoc* and characterised as hypothesis-generating. The signal is nonetheless consistent with methylprednisolone acting as a pharmacological amplifier of adrenergic sensitivity rather than as an independent vasopressor. A receptor-level mechanism plausibly underlies this amplification. Glucocorticoids exert a permissive effect on catecholamine signalling by upregulating adrenergic receptor transcription and replenishing the receptor pool depleted through agonist-induced downregulation. They thereby counteract the desensitisation described above (20). Direct clinical support comes from a study in shock patients receiving prolonged catecholamine infusion. In that study an intravenous methylprednisolone bolus attenuated the catecholamine-induced reduction in myocardial *β*-adrenergic receptor density, and this was accompanied by improvements in cardiac output and blood pressure. The receptor-density effect was demonstrated in the myocardium, whereas the net haemodynamic separation in STEROHCA localised to the vasculature. Restored adrenergic responsiveness may therefore manifest predominantly as attenuated vasoplegia in the post-arrest setting rather than as enhanced contractility. Meta-analytic evidence supports this interpretation, as neurological outcome and survival benefits emerge specifically when corticosteroids are combined with vasopressors rather than administered alone (21). Endothelial protective effects provide a complementary rationale for the intra-arrest timing, including preservation of glycocalyx integrity under ischaemia-reperfusion conditions (22).

The second time window opens after ROSC. The pharmacological target shifts from haemodynamic rescue to endocrine reconstitution. A substantial proportion of post-arrest patients develop critical illness-related corticosteroid insufficiency (CIRCI). This state is characterised by impaired corticotropin-releasing hormone signalling, attenuated adrenocorticotrophic hormone secretion, and peripheral glucocorticoid resistance (23). Empirical hydrocortisone administration without prior stimulation testing is supported by evidence that the diagnostic test fails to reliably identify adrenal insufficiency in acute critical illness. The same test may augment cytokine production in the process (24). This finding was generated in a sepsis population, but the endocrine physiology of CIRCI is shared across critical illness states. Post-ROSC hydrocortisone therefore targets a clinically distinct and pharmacologically separable problem from intra-arrest methylprednisolone. This two-phase steroid strategy constitutes one of the defining features of the full Mentzelopoulos protocol.

### 2.4 Mechanistic predictions for clinical outcomes

**Figure 3:**
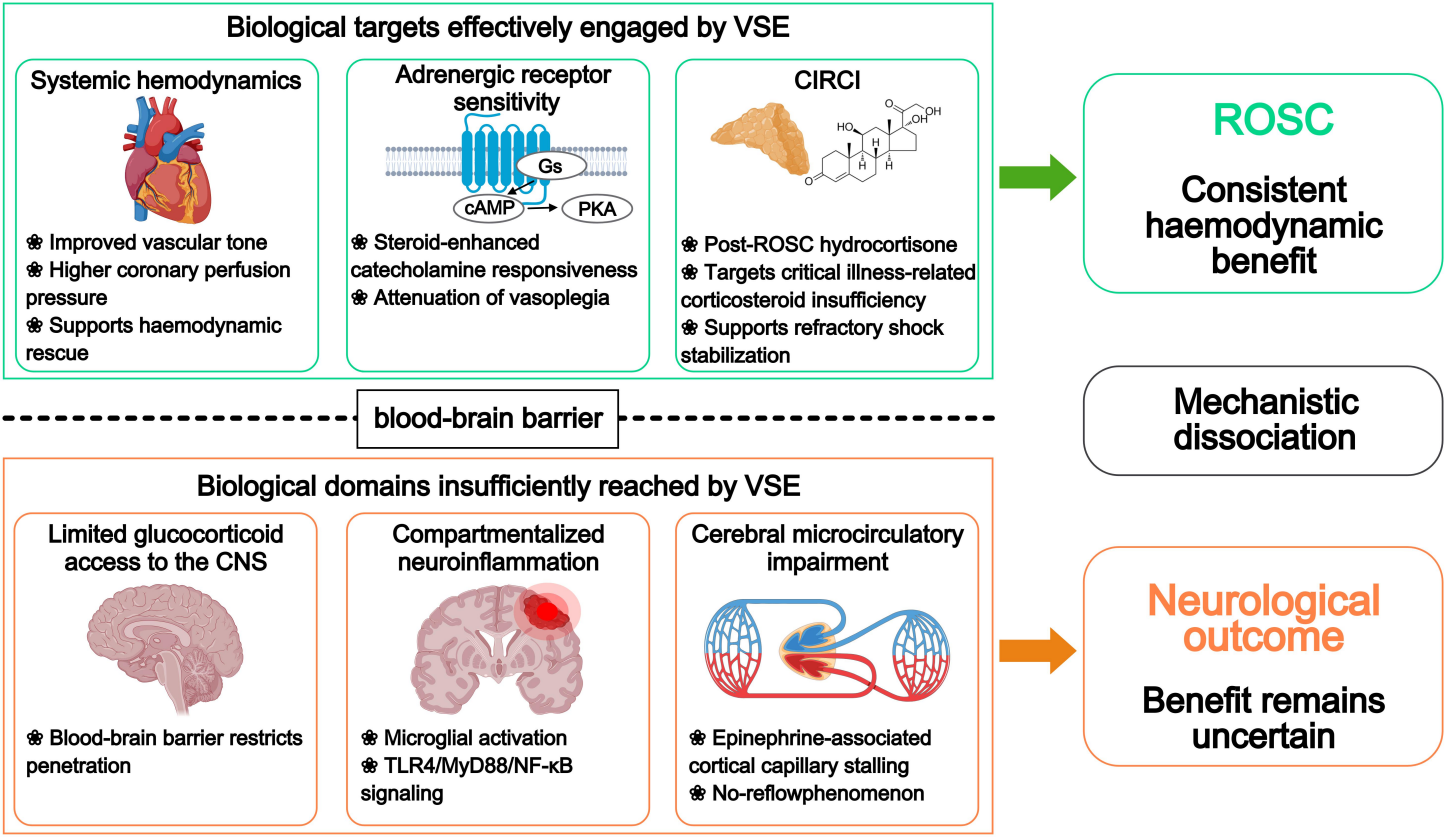
Biological framework explaining the dissociation between ROSC and neurological outcome under VSE therapy. VSE supports ROSC through three engaged targets, namely systemic haemodynamics, adrenergic receptor sensitivity, and CIRCI. Three domains remain insufficiently reached, namely glucocorticoid access to the CNS, compartmentalised neuroinflammation, and epinephrine-associated cerebral microcirculatory impairment. This biological separation helps explain why ROSC benefit does not consistently translate into favourable neurological outcome.

The mechanistic architecture described above carries an important implication for clinical outcomes. The VSE combination addresses the acute haemodynamic failure that prevents ROSC through V1a-mediated vasoconstriction and adrenergic sensitisation. Neurological recovery depends on a different set of biological processes. These processes operate beyond the therapeutic reach of the regimen, and they are summarised in Figure 3**.**

VSE engages three biological targets effectively. Systemic haemodynamics improve through enhanced vascular tone and coronary perfusion pressure. Adrenergic receptor sensitivity is partially restored through steroid-mediated catecholamine responsiveness. CIRCI is addressed through post-ROSC hydrocortisone. Three additional domains remain insufficiently engaged. Circulating glucocorticoids reach the central nervous system only partially. Their access is constrained by blood-brain barrier penetration limits and a restricted capacity to suppress resident microglial activation. Microglial M1 polarisation driven by TLR4/MyD88/NF-*κ*B signalling represents one neuroinflammatory programme activated locally within the CNS following arrest (25). This programme drives the downstream release of pro-inflammatory cytokines, principally TNF-α and IL-6. These cytokines propagate injury by disrupting blood-brain barrier integrity, amplifying glial activation, and promoting secondary neuronal death. Because these mediators are generated within the CNS compartment, they lie largely beyond the reach of circulating glucocorticoids. Epinephrine itself promotes cortical capillary stalling and cerebral no-reflow in the early post-ROSC period, as demonstrated in a porcine model using *in vivo* multiphoton microscopy (26). The drug most effective at restoring cardiac output may simultaneously impair the cerebral microcirculatory reperfusion that neurological recovery requires. This tension cannot be resolved by either vasopressin or corticosteroids within VSE.

Direct experimental support for this dissociation comes from a porcine randomised study showing that targeting higher mean arterial pressure after resuscitation increased cerebral blood flow while simultaneously elevating hippocampal concentrations of TNF-α, IL-6, and lipocalin-2 (27). Haemodynamic rescue and neuroinflammatory suppression are not parallel processes. The clinical evidence examined in the following analysis bears out the predicted divergence between ROSC gains and neurologically intact survival.

## 3 Clinical evidence

### 3.1 Foundational randomised controlled trial evidence

The evidentiary foundation for VSE in IHCA rests on three randomised controlled trials, summarised in Table 1. The first, a pilot trial at a single Greek tertiary care centre, enrolled 100 patients and reported significant improvements in both ROSC and survival to discharge with favourable neurological outcome, establishing biological plausibility for the combination approach (9). The confirmatory trial enrolled 268 patients across three Greek tertiary care centres and demonstrated significantly higher rates of ROSC and neurologically favourable survival (8). The wide confidence interval around the favourable neurological survival estimate arose from only 25 outcome events among 268 patients, reflecting statistical power insufficient to draw reliable conclusions about neurological recovery as a standalone endpoint. Both Greek trials were conducted within a single healthcare system, limiting generalisability.

**Table 1:** Design and outcomes of the three randomised controlled trials evaluating VSE in in-hospital cardiac arrest.

Variable	Mentzelopoulos 2009	Mentzelopoulos 2013	VAM-IHCA 2021/2022
Trial design	Single-centre, RDBPC	Multicentre (3 sites), RDBPC	Multicentre (10 sites), RDBPC superiority
Country	Greece	Greece	Denmark
Sample size (intervention/control)	100 (48/52)	268 (130/138)	501 (237/264)
Intra-arrest vasopressin	20 IU/CPR cycle, max 5 cycles	20 IU/CPR cycle, max 5 cycles	20 IU/epinephrine dose, max 4 doses
Intra-arrest methylprednisolone	40 mg once (first cycle)	40 mg once (first cycle)	40 mg once
Post-ROSC hydrocortisone	Yes (300 mg/day, ≤7 days, tapered)	Yes (300 mg/day, ≤7 days, tapered)	No (omitted)
Protocol completeness	Full VSE protocol	Full VSE protocol	Truncated protocol
Primary endpoint	ROSC ≥15 min + survival to discharge	ROSC ≥20 min + survival to discharge with CPC 1–2	ROSC ≥20 min
ROSC	81% vs 52%; *P* = 0.003	83.9% vs 65.9%; OR 2.98 (95% CI 1.39–6.40)	42% vs 33%; RR 1.30 (95% CI 1.03–1.63)
Survival to discharge/30-day survival	19% vs 4%; *P* = 0.02	Not reported separately	9.7% vs 12.0%; RR 0.83 (95% CI 0.50–1.37)
Favourable neurological outcome (CPC 1–2)	Not assessed	13.9% vs 5.1%; OR 3.28 (95% CI 1.17–9.20)	7.6% vs 7.6%; RR 1.00 (95% CI 0.55–1.83)
1-year survival	Not reported	Not reported	6.3% vs 8.3%; RR 0.76 (95% CI 0.41–1.41)
1-year favourable neurological outcome	Not reported	Not reported	5.9% vs 7.6%; RR 0.78 (95% CI 0.41–1.49)

CPC, cerebral performance category; OR, odds ratio; RDBPC, randomised double-blind placebo-controlled; RR, risk ratio; ROSC, return of spontaneous circulation; VSE, vasopressin-steroids-epinephrine.

The multicentre VAM-IHCA trial was designed to replicate the ROSC benefit at greater scale. Across ten Danish hospitals and 501 patients, it confirmed a statistically significant improvement in ROSC, but 30-day survival and favourable neurological outcome did not differ between groups (10). The null pattern persisted at one-year follow-up (11). Three interrelated concerns shape interpretation of this divergence. First, VAM-IHCA has been argued to have been adequately powered for ROSC but substantially underpowered for 30-day survival or neurological outcome (28). Second, the trial omitted the post-ROSC stress-dose hydrocortisone continuation that was a defining feature of the original Mentzelopoulos protocol (29, 30). Third, VAM-IHCA tested only the intra-arrest vasopressin and methylprednisolone components, evaluating a truncated version of the full regimen. The trial authors acknowledged that VAM-IHCA was not designed to replicate the complete Mentzelopoulos protocol, and that the omission of post-ROSC steroids may have constrained its capacity to detect downstream effects on survival and neurological outcome (31). A 2024 reappraisal in Critical Care argued that this omission constitutes a substantive departure from the original protocol rather than a faithful replication (32).

### 3.2 Meta-analyses and network meta-analyses

Pooled analyses of the available randomised evidence have consistently reproduced the ROSC signal while failing to establish equivalent confidence in survival and neurological endpoints. An umbrella review applying AMSTAR-2 criteria to 21 eligible systematic reviews retained twelve for substantive analysis, across which the direction of effect for ROSC was uniform (33). The principal meta-analytic evidence is summarised in Table 2.

**Table 2:** Meta-analytic evidence evaluating VSE and corticosteroid-based interventions in cardiac arrest.

Meta-analysis	Trials (n)/Patients (*N*)	Intervention scope	ROSC effect (95% CI)	Survival/neurological outcome effect (95% CI)	GRADE certainty
Holmberg et al., 2022 (34)(IPD meta-analysis)	3 RCTs/*N* = 869	VSE combination (IHCA only)	OR 2.09 (1.54–2.84)	Survival to discharge: OR 1.39 (0.90–2.14); favourable neurological outcome: OR 1.64 (0.99–2.72)	ROSC: moderate; survival and neurological outcome: low
Zhou et al., 2024 (38)	11 RCTs/*N* = 2,273	Corticosteroid monotherapy (IHCA and OHCA)	OR 2.05 (1.24–3.37)	Favourable neurological outcome: OR 1.13 (0.81–1.58); survival to discharge: OR 1.29 (0.74–2.26)	Not reported
Saghafi et al., 2026 (36) (network meta-analysis)	36 RCTs/*N* = 21,768	VSE vs monotherapy and dual-therapy comparators (IHCA and OHCA mixed)	VSE ranked highest across all comparators for ROSC, survival to admission, 24-hour survival, and survival to discharge	Neurological outcomes not pooled (measurement heterogeneity across trials)	Not reported
Dhingra et al., 2026 (41)	11 studies/*N* = 2,605	Immunomodulatory interventions post-ROSC (corticosteroids 82%, IL-6 receptor blockade 9%, calcineurin inhibition 9%, IHCA and OHCA mixed)	Not applicable (post-ROSC interventions only)	Favourable neurological outcome RR 1.04 (0.85–1.28). 30-day mortality RR 0.97 (0.91–1.03)	Not reported
Penn et al., 2023 (29)	8 RCTs/*N* = 2,213	Corticosteroid administration in cardiac arrest (IHCA and OHCA mixed)	RR 1.32 (1.18–1.47), derived from 4 trials and 919 patients	Mortality RR 0.96 (0.90–1.02). Favourable neurological outcome RR 1.08 (0.94–1.25)	ROSC moderate. Survival and neurological outcome low

CI, confidence interval; GRADE, Grading of Recommendations Assessment, Development and Evaluation; IHCA, in-hospital cardiac arrest; IPD, individual participant data; OHCA, out-of-hospital cardiac arrest; OR, odds ratio; RCT, randomised controlled trial; ROSC, return of spontaneous circulation; RR, risk ratio; VSE, vasopressin-steroids-epinephrine.

The individual participant data meta-analysis pooling all three foundational trials provides the most methodologically rigorous estimate currently available, yielding a moderate-certainty GRADE estimate for ROSC and low-certainty estimates for both survival to discharge and favourable neurological outcome (34). A subsequent corrigendum strengthened the directional signal for favourable neurological outcome without altering the overall conclusion (35). This stratification of certainty across endpoints reflects that the evidence base was powered primarily to detect ROSC differences and that neurological outcome events were too infrequent across trials to support confident inference.

The most current network meta-analytic synthesis expands this picture. A network meta-analysis of 36 randomised trials enrolling 21,768 patients ranked VSE above all monotherapy and dual-therapy comparators across ROSC, survival to admission, 24-hour survival, and survival to discharge (36). Neurological outcomes were not pooled owing to measurement heterogeneity. The ranking conclusion therefore pertains to haemodynamic and survival endpoints without resolving the neurological recovery question. The included trials are heterogeneous in setting, mixing IHCA and out-of-hospital populations, and the largest single IHCA replication trial was neutral for survival, features that temper interpretation of the ranking finding (37).

The corticosteroid component receives independent support from two convergent meta-analyses. Zhou and colleagues found that corticosteroid administration significantly improved ROSC without producing significant improvement in favourable neurological outcome or survival to discharge (38). Penn and colleagues reported a moderate-certainty ROSC benefit under similar null findings for survival and neurological outcome (39). The convergence between these estimates on the direction of effect for ROSC, despite differences in trial inclusion and effect measures, strengthens confidence in the haemodynamic signal while reinforcing the persistent null pattern for patient-centred endpoints.

Alongside this broadly positive ROSC signal, the CORTICA trial enrolled 184 IHCA patients and compared a lower intra-arrest methylprednisolone dose of 40 mg combined with post-ROSC hydrocortisone against placebo (40). All haemodynamic and clinical endpoints were negative. Convergent evidence from a *post-hoc* haemodynamic analysis of VAM-IHCA reinforces this interpretation, with the 40 mg dose producing no significant between-group differences in mean arterial pressure, vasopressor requirements, or arterial blood gas parameters within the first 24 h after ROSC (37). Taken together, these data suggest the vasopressor-sparing signal observed with high-dose methylprednisolone in STEROHCA may be dose-dependent, and that trials employing lower steroid doses may be testing a pharmacologically distinct intervention rather than a faithful replication of the original protocol.

A complementary perspective comes from a meta-analysis of eleven studies enrolling 2,605 post-arrest patients, which examined the broader landscape of immunomodulatory interventions in the post-resuscitation period (41). The included interventions comprised corticosteroids in approximately 82% of cases, with interleukin-6 receptor blockade and calcineurin inhibition accounting for the remainder. Near-unity estimates for both favourable neurological outcome and 30-day mortality across this larger and more heterogeneous evidence base indicate that broad anti-inflammatory strategies in the post-resuscitation phase have not yet translated into measurable gains in neurological recovery or survival.

### 3.3 Individual patient data analysis

A pooled reanalysis of individual patient data from the two Mentzelopoulos trials offers the most granular evidence currently available for examining the contributions of the constituent drugs (42). Among the 191 patients who developed post-resuscitation shock across both trials, exposure to stress-dose corticosteroids was associated with a significantly lower risk of lethal septic shock, with a cause-specific hazard ratio of 0.40 (95% CI 0.20–0.82) in the as-treated analysis. The association remained robust after adjustment for baseline differences in arrest aetiology, suggesting that the corticosteroid component carries independent protective value in the post-resuscitation period beyond the vasopressor effects of epinephrine and vasopressin.

Adjustment for arrest aetiology treats it as a confounder, yet aetiology may also act as an effect modifier. The magnitude of benefit from VSE could therefore differ across the underlying causes of arrest. Several mechanistic considerations make this plausible. Arrests with a vasoplegic or septic component, or with coexisting relative adrenocortical insufficiency, might respond more strongly to the corticosteroid and vasopressin components. This expectation aligns with the protective signal observed in the post-resuscitation shock subgroup above. Arrests of primary cardiac origin, such as ischaemic arrhythmic events, might instead depend chiefly on coronary perfusion and adrenergic support, where the added value of the two non-catecholamine components could be smaller. Presenting rhythm and arrest duration represent related dimensions, given their links to the degree of adrenergic receptor downregulation described earlier.

Methodological constraints warrant careful consideration. The dataset derived from two trials not originally designed with a factorial structure. The contribution of each individual drug was therefore never tested in isolation across the full cohort. The analytic framework incorporated both intention-to-treat and as-treated approaches. The latter was introduced partly because fifteen control patients in the 2013 trial received open-label corticosteroids at their treating physicians' discretion. The effective sample size of 191 patients produced wide confidence intervals around statistically significant hazard ratio estimates. These constraints frame the finding as a signal warranting prospective investigation rather than as confirmatory evidence of a causal effect. The same constraints preclude any reliable aetiology-stratified interaction analysis, so effect modification by arrest cause remains hypothesis-generating. No completed trial has employed a factorial design capable of isolating the independent contribution of each drug within the combination. This gap remains one of the most important structural limitations of the current evidence base.

The evidence reviewed across randomised trials and individual patient data analyses points toward a clinical application logic that is both phase-specific and phenotype-specific. Vasopressin and intra-arrest methylprednisolone address acute haemodynamic failure during CPR, and the evidence supporting this phase carries the highest certainty in the literature. Post-ROSC hydrocortisone targets a pharmacologically separable problem, namely corticosteroid insufficiency and vasopressor dependence rather than circulatory arrest itself. The omission of post-ROSC hydrocortisone in VAM-IHCA represents the single most consequential protocol difference between the trials demonstrating neurological benefit and the trial that did not. Beyond the temporal dimension, the independent protective signal for the corticosteroid component against post-resuscitation septic shock suggests that this subgroup may represent the population in which the complete Mentzelopoulos protocol carries the most clinically defensible rationale.

### 3.4 Extension to out-of-hospital settings

Whether VSE can produce comparable benefits in out-of-hospital cardiac arrest (OHCA) represents the current frontier of this evidence base. The VAST-A randomised pilot study, conducted across three Swedish hospitals, randomised 39 of 183 screened IHCA patients to VSE or placebo, with ROSC achieved more frequently in the VSE group (43). The authors concluded that randomisation was feasible from a safety standpoint but that enrolment rates would require improvement in any definitive trial. The directionally positive ROSC signal is consistent with the broader literature, though the sample precludes inference about survival or neurological outcomes.

A multicentre cluster-randomised superiority trial, OHCA REVIVES, has been designed to evaluate the full VSE combination during prehospital resuscitation across six advanced ambulance teams in Taiwan, with a planned enrolment of 1,344 patients (44). The primary endpoint is sustained ROSC lasting at least two hours, a more stringent threshold than the twenty-minute criterion used in the 2013 Mentzelopoulos trial. Several features of the OHCA context introduce design challenges. Cluster-level allocation reduces logistical barriers but introduces potential for systematic imbalance in baseline arrest characteristics across allocation periods. The logistical constraints of prehospital care also create uncertainty about whether post-ROSC hydrocortisone continuation can be delivered with equivalent fidelity during ambulance transport or emergency department handover. Several operational measures could mitigate these barriers. Dedicated pre-packaged VSE emergency kits, containing pre-labelled agents together with a printed dosing algorithm, would shorten preparation time and reduce dosing errors during a time-critical resuscitation. Standardised handover checklists for the ambulance to emergency department transition would help ensure that post-ROSC hydrocortisone is reliably initiated and that previously administered drugs are accurately communicated. Embedding these tools within existing resuscitation quality-improvement and simulation-based training frameworks would further support protocol fidelity in future OHCA trials. The OHCA REVIVES results will determine whether the mechanistic logic underpinning VSE translates across the operational and biological differences separating prehospital from in-hospital resuscitation.

## 4 Dissociation between ROSC and neurological outcome, and safety considerations

The moderate-certainty ROSC benefit of VSE stands in consistent contrast to the low-certainty estimates for survival to discharge and favourable neurological outcome, a pattern that holds across both the individual participant data meta-analysis and the broader immunomodulatory literature (34, 41). Three factors account for this dissociation, each operating at a different level of analysis.

The first factor is methodological. The available randomised evidence was powered to detect ROSC differences rather than neurological outcomes, and the largest IHCA replication trial evaluated a truncated version of the full protocol, as discussed in the preceding clinical evidence analysis. The second factor concerns variability in post-resuscitation care. Differences in targeted temperature management, haemodynamic targets, and ventilatory strategy across trial sites introduce heterogeneity in the neurological recovery environment that no intra-arrest pharmacological intervention can control. This phase of care is independently determinative of neurological trajectory and cannot be treated as a neutral background variable when attributing outcome differences to intra-arrest drug effects. The third factor is biological, and it is the most fundamental. As established in the pharmacological framework above, circulating glucocorticoids reach the neuroinflammatory compartment only partially. Epinephrine itself promotes cortical capillary stalling in the early post-ROSC period. Human data from the STEROHCA trial confirm that substantial suppression of systemic IL-6 following high-dose glucocorticoid administration produces no detectable change in neuron-specific enolase, indicating that systemic anti-inflammatory efficacy and neuronal injury mitigation remain biologically distinct events (45).

Taken together, these three factors predict from first principles that gains in ROSC and gains in neurologically intact survival should diverge under VSE. This prediction is borne out with notable consistency across the clinical evidence. The position of international resuscitation guidance reflects this evidentiary pattern. The 2025 American Heart Association advanced life support guideline rates vasopressin as offering no advantage over epinephrine, whether used alone or in combination, and assigns intra-arrest corticosteroid administration a Class 2b designation indicating uncertain benefit (46). The 2025 European Resuscitation Council guideline reaches a similar position and does not recommend routine VSE pending replication (47). The 2025 International Liaison Committee on Resuscitation Consensus on Science with Treatment Recommendations notes that new analyses of vasopressin and methylprednisolone added to standard care show no difference in haemodynamics or long-term outcomes (48). No major resuscitation society currently recommends routine VSE during in-hospital cardiac arrest.

The available evidence does not identify a consistent pattern of serious adverse effects attributable to VSE when administered according to the Mentzelopoulos protocol. The VAST-A randomised pilot study reported no unexpected adverse events in the VSE group, supporting the feasibility of intra-arrest administration from a safety standpoint (43). Vasopressin carries a dose-dependent risk of V2 receptor-mediated renal water reabsorption and augmented capillary permeability, though these effects are most relevant in the context of prolonged infusion rather than the time-limited intra-arrest bolus dosing used in the major VSE trials. The theoretical concern that intra-arrest corticosteroids might predispose survivors to post-resuscitation infection has not been confirmed. The individual patient data reanalysis of the two Mentzelopoulos trials found that stress-dose corticosteroid exposure was associated with a significantly lower incidence of lethal septic shock, suggesting that glucocorticoid immunomodulation in the post-arrest period does not operate through straightforward immunosuppression (42). Post-ROSC hydrocortisone at stress doses is consistent with established practice in vasopressor-dependent critical illness. Definitive safety characterisation remains contingent on the larger patient-centred endpoint data anticipated from the OHCA REVIVES trial.

The scale of trial required to resolve these questions can be approximated directly. As noted earlier, the available evidence was powered for ROSC rather than for favourable neurological outcome. A useful benchmark is the ability to detect an absolute improvement of about five percentage points in favourable neurological survival. Assuming a control rate near 10 percent, a two-sided alpha of 0.05, and 80 percent power, roughly 680 patients per arm would be needed, or approximately 1,400 in total. Detecting a smaller and arguably more realistic effect of three to four percentage points would require several thousand patients, on the order of 2,800–4,100. These figures should be inflated by a further 10–15 percent to allow for withdrawal and crossover. The estimates are approximate and depend on the assumed baseline rate and effect size, and any definitive calculation should be fixed by a trial statistician. Even so, they show why the single-centre and moderately sized trials conducted to date were necessarily underpowered for neurological endpoints.

## 5 Conclusion

The VSE combination produces a reliable improvement in ROSC, supported by convergent evidence across randomised trials, individual participant data analyses, and network meta-analyses. This haemodynamic benefit follows from a pharmacological design that addresses limitations epinephrine monotherapy cannot overcome during prolonged resuscitation. The translation of this advantage into neurologically intact survival has not been reliably demonstrated. This failure reflects the boundaries of what circulating agents can reach rather than an absence of biological activity. Compartmentalised neuroinflammatory processes and epinephrine-mediated cerebral microcirculatory impairment lie beyond the therapeutic range of the current regimen.

Two priorities follow from this evidence. The independent protective signal for the corticosteroid component against post-resuscitation septic shock provides a plausible rationale for applying the complete Mentzelopoulos protocol in IHCA patients with refractory shock, pending guideline endorsement. Beyond this application, patient phenotype stratification and a prospectively designed factorial trial represent the most tractable paths toward isolating the contribution of individual drugs and generating patient-centred endpoint evidence that current syntheses cannot deliver. Advancing outcomes in cardiac arrest will ultimately depend on recognising that restoring spontaneous circulation and preserving neurological function are biologically distinct therapeutic goals, each requiring its own dedicated pharmacological strategy.
